# Survey of bed bug infestations in homeless shelters in southern France

**DOI:** 10.1038/s41598-023-38458-2

**Published:** 2023-08-02

**Authors:** Bouthaina Hasnaoui, Jean Michel Bérenger, Pascal Delaunay, Adama Zan Diarra, El Hadji Ibrahima Ndiaye, Saidou Ahamada M’madi, Noelle Masotti, Jacques Sevestre, Philippe Parola

**Affiliations:** 1 Aix Marseille Univ, IRD, SSA, AP-HM, VITROME, 19-21 Boulevard Jean Moulin, 13005 Marseille, France; 2 IHU-Méditerranée Infection, Marseille, France; 3 Laboratory of Parasitology Mycology, Nice University Hospital, Nice, France

**Keywords:** Health care, Zoology, Entomology

## Abstract

Bed bug has become a major public health pest worldwide. Infestation may result in numerous negative health effects. Homeless shelters are one of the most habitats that can be infested with bed bugs, a few studies have focused on bed bug infestations in these settings. We conducted a survey of infestations of bed bugs in a homeless shelter in southern France, using an innovative seven-level scale (0–6) to assess the degree of infestation, MALDI TOF-MS to identify bed bugs, and a biomolecular tool to detect bacteria. Bed bug infestations were documented in 13% (9/68) of investigated rooms. A total of 184 bed bugs were collected and morphologically identified as *Cimex lectularius*. MALDI TOF–MS analysis allowed us to obtain high-quality MS spectra for all 184 specimens, to correctly identify all specimens, and included 178/184 (97%) Log Score Values higher than 1.8. Among the bacteria tested, *Wolbachia* sp. DNA was found in 149/184 (81%) of the bed bugs, and one sample was positive for *Coxiella burnetii*, the agent of Q fever. Our study is the first of its kind that offers new perspectives for increasing public awareness of the conditions in homeless shelters.

## Introduction

By their nature, homeless shelters are at risk of infestation by numerous obligate hematophagous arthropods including body lice, mites, and bed bugs^1,2^. Bed bugs are flat brown insects, and all developmental stages are hematophagous^3–5^. Previous research has shown that bed bug infestations are spreading throughout the world, notably in refugee camps and homeless shelters, which offer favourable conditions for their development^3^.

*Cimex lectularius*, the common bed bug, *C. hemipterus*, the tropical bed bug, and *C.adjunctus,* bat bugs, are the three main species for which infestations in human habitations have been documented^3,6^. *Cimex lectularius* may also feed on other animals such as bats and birds^5,7,8^. Most recently, *C. hemipterus* has been observed in Europe, where *C. lectularius* is a well-established species^9,10^. Surveys are, therefore, crucial to monitor the distribution of different species in real time.

The mouthparts of bed bugs are designed to pierce the skin and suck blood^2^. These parts include the mandibular and maxillary stylets which enable the injection of saliva, a crucial immunogenic component, as reported in recent studies focusing on dermatological and immuno-allergic^11^ reactions to bites^12^. Bed bug infestations may have psychological consequences and it has been proven that bed bugs make people fearful^3,13^. Additionally, some cases of infestation have been linked to anaemia, an iron deficiency^14^.

Currently there is no proof that bed bugs may transmit infectious agents to humans in “real life”^3,15^. However, over 40 potential human pathogens have been detected in the blood meals of bed bugs. Recent experimental studies have also suggested their vectorial competence for pathogens such as *Bartonella quintana, Borrelia reccurentis* and *Trypanosoma cruzi*^16,17^. In a separate study, researchers examined bed bugs collected from a homeless shelter and discovered the presence of gram-positive bacteria in *Cimex lectularius* specimens^18^.

The habit that bed bugs have of concealing themselves may impede early detection of an infestation, adding to the difficulty of eradication^19,20^. The early developmental stages of bed bugs lead to them being commonly mistaken for other arthropods (cockroaches, *Anthrenus verbasci* larvae, etc.). Identifying *Cimex* species is not an easy task with morphological and molecular techniques, which are consuming and expensive^21^. Mass spectrometry (MALDI-TOF MS) has recently been successfully used to identify laboratory-reared bed bugs as well as wild bed bugs collected in the field in Africa were it showed its efficacity as an alternative method to identify and discriminate *Cimex* species^22^.

This study has two parts: field and laboratory. We have first investigate a homeless shelter in France to evaluate the infestation level using an innovative scale, collect the bed bugs and give advice for pest control for home staff. In a second time, specimens collected were identified using MALDI TOF-MS in conjunction with morphological and molecular analysis and then screening for associated microorganisms.

## Results

### Collection site and morphological identification of bed bugs

**Figure 1:**
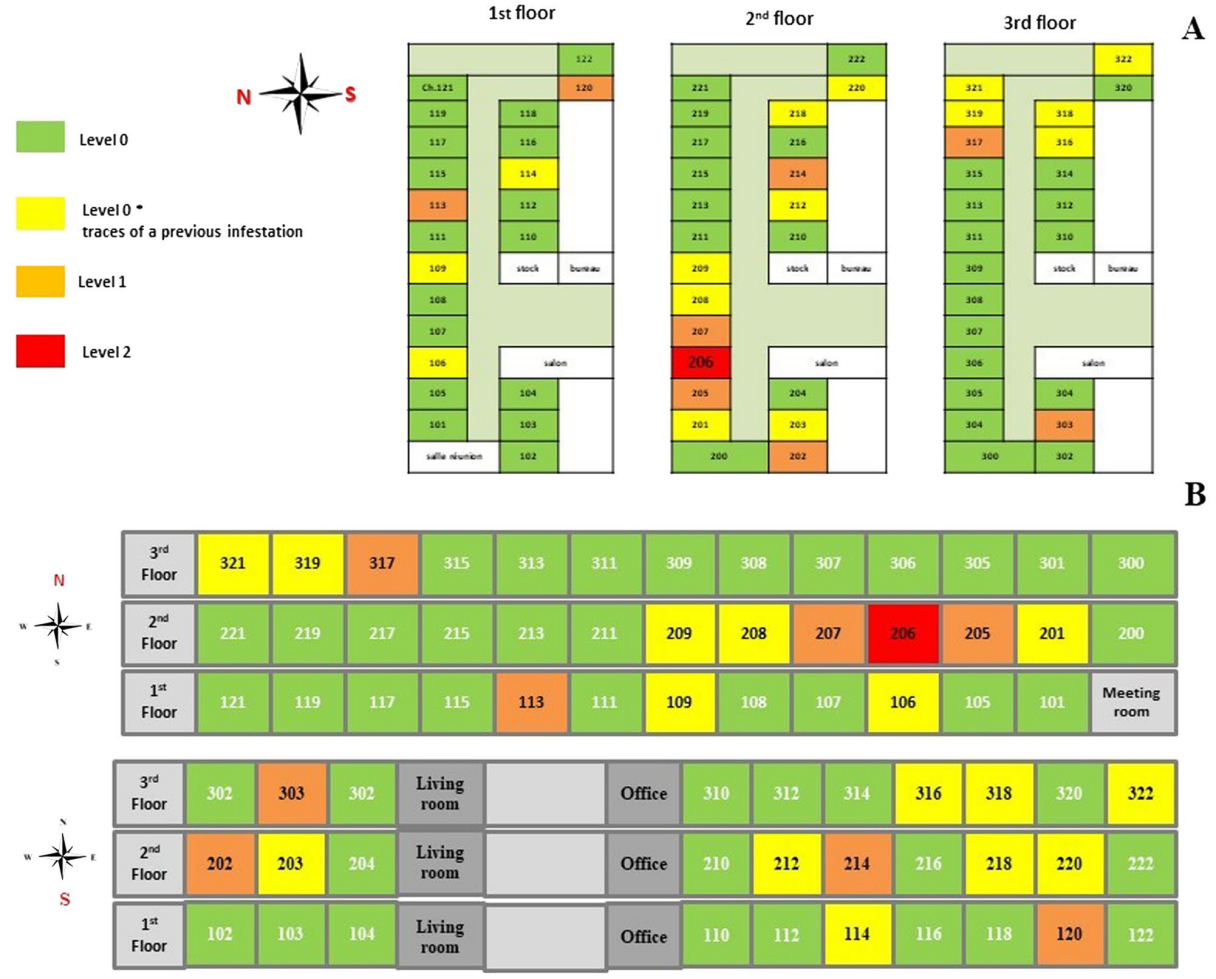
A summary of each floor and the bed bug-infested rooms (**A**), the north and south sides each floor are superimposed to show the extent of the bed bug infestation (**B**).

Based on the initial signs of a bed bug infestation (dark faecal spots), 68 rooms were inspected; 35% (24/68) rooms had signs of infestations (dark faecal spots), of which 63% (15/24) were linked to a previous infestation. Only 37% (9/24) were found to be positive for bed bugs. The infestation levels in the rooms ranged from level 0 to level 2, and the level of infestation in the building was level 4 (Fig. 1).

A total of 184 bed bugs were collected from the positive rooms. The locations of the bed bugs and the level of infestation, as well as the distribution of bed bugs among the infested rooms are summarised in Table 1. Bed bugs were divided into different stage: immatures and adults (male or female) and engorged or not engorged according to their morphology and morphometry. A total of 119 immature bed bugs, 40 females and 25 males, were identified. All specimens were identified as *C. lectularius.* Supplementary 3 shows images of the morphological traits taken with a Canon camera.

**Table 1:** Sampling bed bugs from a room in the shelter.

Room number	Evidence of bed bug infestation (dark fecal spots)			Presence of bedbugs and collection					Infestation level
	Beds	Mattress	Walls	Beds	Mattress	Walls	Wheelchair	Nb	
106°	+	+	−	− −	− −	− −	− −	0	Level 0*
109°	+	+	−	− −	− −	− −	− −	0	Level 0*
113°	+	+	−	+ +	− −	− −	− −	5	Level 1
114°	+	+	−	− −	− −	− −	− −	0	Level 0*
120°	+	+	−	+ +	− −	− −	− −	7	Level 1
201°	+	+	−	− −	− −	− −	− −	0	Level 0*
202	−	−	−	− −	− −	− −	+ +	20	Level 1
203°	+	+	−	− −	− −	− −	− −	0	Level 0*
205	+	+	−	+ +	− −	− −	− −	24	Level 1
206°	+	+	+	+ +	+ +	+ +	− −	90	Level 2
207	+	+	−	+ +	− −	− −	− −	9	Level 1
208°	+	+	−	− −	− −	− −	− −	0	Level 0*
212°	+	+	−	− −	− −	− −	− −	0	Level 0*
214	+	+	−	+ +	− −	− −	− −	3	Level 1
218°	+	+	−	− −	− −	− −	– –	0	Level 0*
303	+	+	−	+ +	− −	− −	− −	6	Level 1
316°	+	+	−	− −	− −	− −	− −	0	Level 0*
317°	+	+	−	+ +	− −	− −	− −	19	Level 1
318°	+	+	−	− −	− −	− −	− −	0	Level 0*
319°	+	+	−	− −	− −	− −	− −	0	Level 0*
321°	+	+	−	− −	− −	− −	− −	0	Level 0*
322°	+	+	−	− −	− −	− −	− −	0	Level 0*

+: Presence of trace of an old infestation.

+  +: Presence of bed bugs.

Nb: Number of specimens collected.

°: Diatom powder treatment = insecticide.

### MALDI TOF–MS bed bug identification

**Figure 2:**
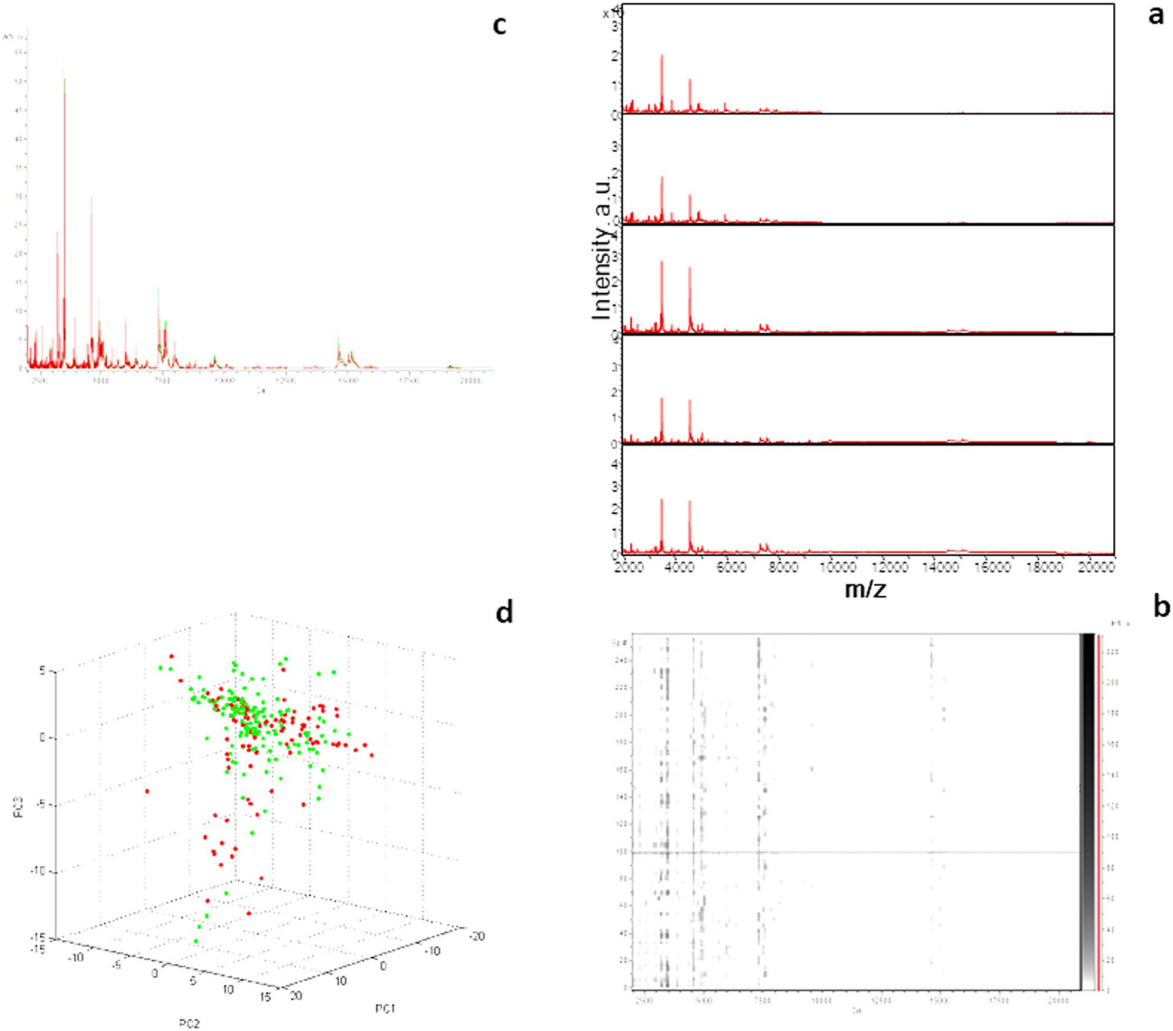
(**a**) MALDI-TOF MS spectra from adult *Cimex lectularius*; (**b**) Gel View representation of MS profiles *Cimex lectularius* adults bedbugs; (**c**) representation and comparison of average male (green) and female (red) MS profiles, superimposed using the clinProTools 2.2 software; (**d**) Principal component analysis (PCA) of male (green) and female (red) MS spectra using clinProTools 2.2 software. a.au = arbitrary units; m/z = mass/charge ratio.

MALDI TOF–MS analysis was performed on the cephalothoraxes of 119 immature samples and the heads of 65 adults. FlexAnalysis v.3.3 software was used to analyse the MS profiles, which revealed that 184 (100%) of the spectra were remarkably reproducible, without background noise, and a smooth baseline for each adult and immature stage analysed. Similar protein patterns were observed in both male and female bed bugs. No discriminating peaks with different intensities could be observed when the MS profiles of the groups of both sexes were superimposed (Fig. 2). Principal component analysis (PCA) was used to statistically compare groups of MS profiles of both sexes, and no differences between the groups were found. The MS spectra from the 184 bed bug specimens were queried against our in-house arthropod MS reference database. All samples were identified as *C. lectularius* by MALDI TOF–MS.

MS profiles from adult head bed bugs (n = 65) were matched with Log Score Values (LSVs) ranging from 1.407 to 2.656, a mean of 2.186 and a median of 2.191, with reference spectra obtained from heads of field-collected adult *C.lectularius.* Among these, 95% (62/65) retrieved an LSV > 1.8, which was considered a reliable identification.

**Figure 3:**
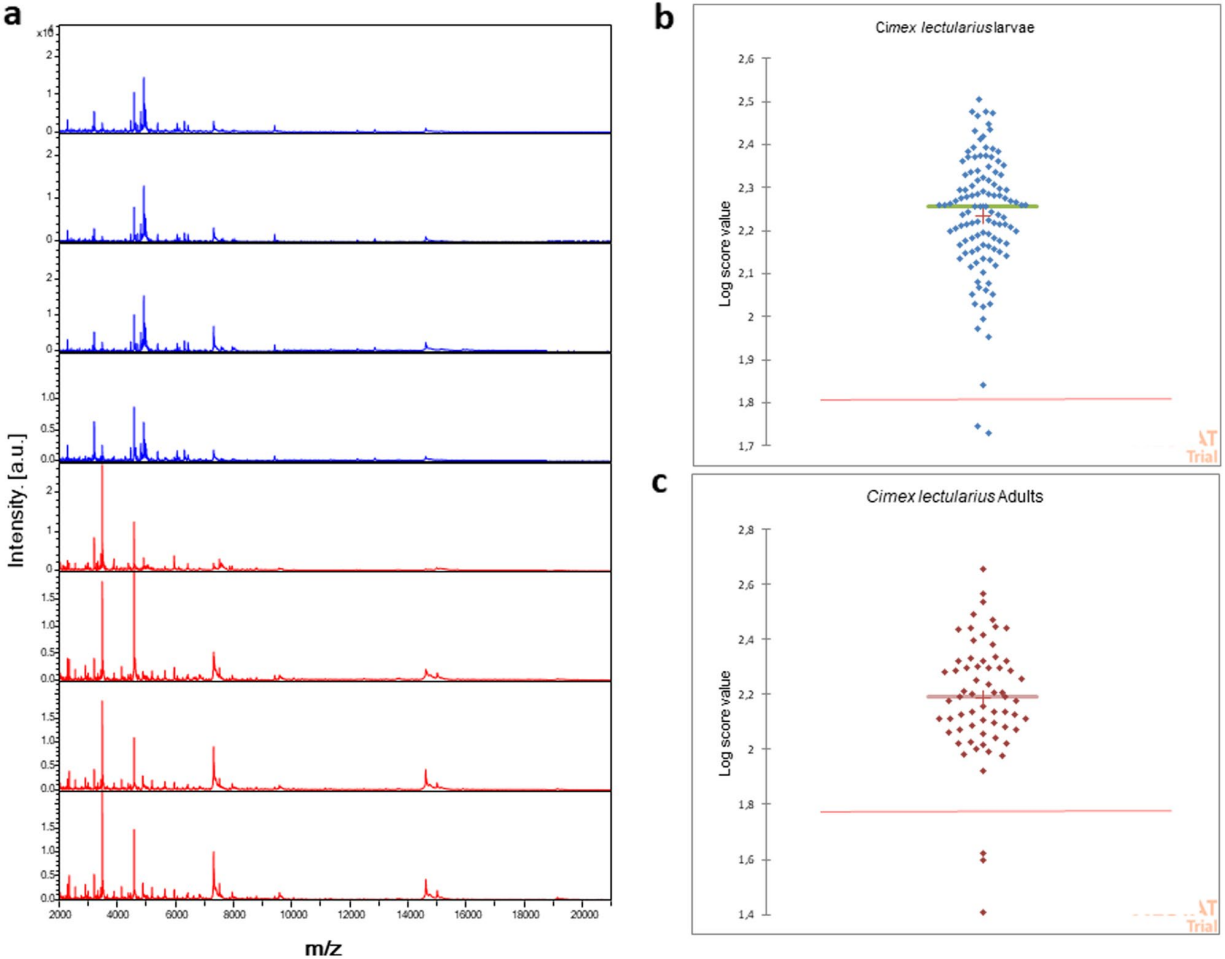
Representative MALDI-TOF MS spectra from heads of adults (red) and cephalothoraxes of larvae of four specimens (blue) of *Cimex lectularius* (**a**); Boxplots showing the log score value (LSV) distribution. The red line represents the threshold of LSV above which the identification is correct, for both adult (red) and larvae (bleu) values (**b**, **c**).

The MS profiles (n = 119) from cephalothoraxes of immature stages matched with LSVs ranging from 1.729 to 2.506, a mean of 2.235 and a median of 2.257, with reference spectra obtained from immature *C. lectularius* cephalothoraxes reared in our laboratory (unpublished data). Of these, 96% (115/119) had an LSV > 1.8. Figure 3 shows representative MALDI TOF–MS spectra from the heads and cephalothoraxes of four specimens of adults and larvae of *C. lectularius,* with a boost plot showing the log score value (LSV) distribution from the 119 *Cimex* immature stages and the 65 adults.

Here, we provide a DOI (https://doi.org/10.35081/zhsq-5905) for a selection of representative spectra of bed bugs randomly selected that has been conducted in this study.

### Molecular detection of microorganisms and phylogenetic analysis

DNA from the 184 bed bugs was screened for the presence of bacteria by qPCR. DNA from bacteria of the *Anaplasmataceae* family was found in 149/184 (81%) of samples using the *23S rRNA* gene. The same positive samples for the *Anaplasmataceae* family were also positive for *Wolbachia spp* using the *16S rRNA* gene. Only one sample was positive for *C. burnetii* using both the *ISS11* and *IS30A* genes, detected in an engorged female bed bug collected in a room with a smaller sample size (n = 5), consisting of one adult engorged female and four non-engorged immature specimens. However, none of the remaining bacteria tested (*Borrelia* spp., *Rickettsia* spp., *Bartonella* spp) in this study could be detected in the DNA of any of the specimens tested.

**Figure 4:**
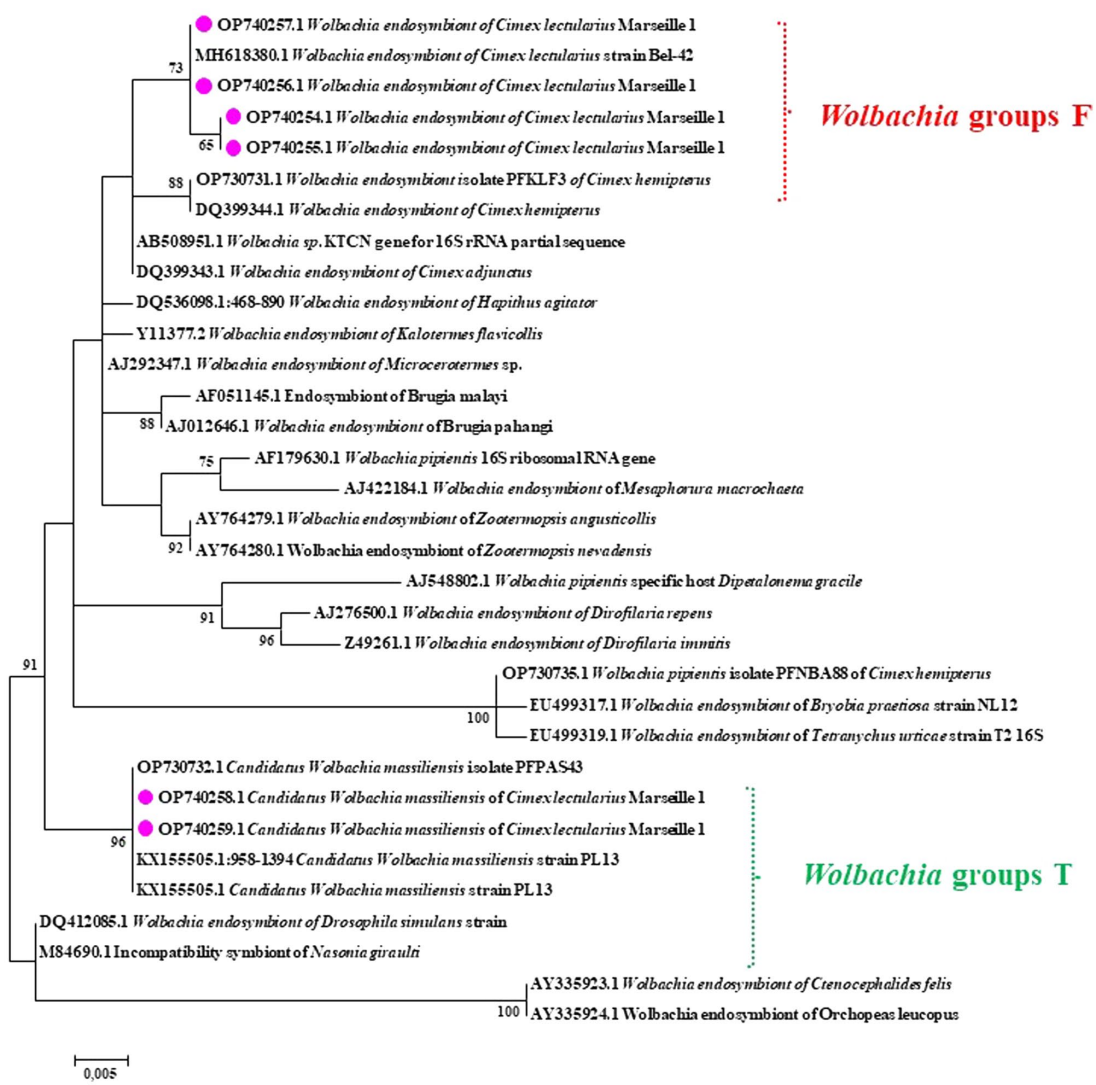
Phylogenetic tree showing the relationships between *Wolbachia* identified in *Cimex lectularius* bed bugs to other species based on a comparison of the sequences of a 356 bp fragment of the *16S rRNA*. The tree was generated using the maximum likelihood algorithm (PhyML) with the symmetrical model (SYM) proposed by the software. The specimens in our study are shown in pink dot, alongside the GenBank accession numbers of each sequence.

In order to define and characterise the *Wolbachia* species found in this study, 25 samples randomly selected from the 149 positive samples were subjected to standard PCR amplification *16S rRNA* (390pb)*.* A total of 19/25 (76%) of the sequences analysed had 99.75–100% similarity with the corresponding sequence of *Wolbachia* endosymbiont (MH618380) found in *C. lectularius* from Serbia^23^. Furthermore, 6/25 (24%) of the sequences had 99.32–100% similarity to the corresponding sequence of candidatus *Wolbachia massiliensis* (CP061738), which was isolated from the tropical *C. hemipterus* from Senegal^23–25^. Our *Wolbachia* sequences belonged to the group F of Fillaria and the group T recently described in *C. hemipterus*, demonstrated in the phylogenetic tree using sequences of *Wolbachia* host species and their affiliation clades among arthropods and nematodes (Fig. 4). Six sequences of the *16S rRNA* of *Wolbachia* obtained in this study were deposited in the GenBank (National Centre for Biotechnology Information, NCBI) under the following accession numbers: OP740254 to OP740259.

### Data metagenomic description

The positive sample for *C. burnetii* processed for sequencing with the Illumina MiSeq has a cluster density of 1469 K/mm^2^ with a cluster passing quality control filters of 83.6%. 11.8 Gb of information was obtained in total. Within this run, the index representation for *C. burnetii* was determined to be 4.9632%. The 2,248,358 paired-end reads were filtered according to the read qualities. The number of reads attributed to bacteria was 13,468; those attributed to viruses, fungi, and protozoa were 438, 701, and 1,050, respectively.

## Discussion

Homeless people are frequently exposed to unclean environments that harbour various hematophagous arthropod species^26,27^. As a result of this exposure, the prevalence of shelters infested by these arthropods is on the rise. There have only been a few cases reported of homeless shelters bed bugs infestations, in comparison to infestations of places with greater economic importance, such as hotels and private housing facilities, which have drawn much greater attention^28^. In this study, we report on the infestation of a French homeless shelter which was investigated after the manager and occupants complained of being bitten by insects and experiencing psychological discomfort.

Studies on the description of the level of infestation generally focus on the quantity of bed bugs present^29,30^, which is a non-discriminating criterion. It is simpler to eradicate bed bugs when they are concentrated in a single, defined area (in this case, the bed). However, it becomes more challenging when bed bugs are dispersed throughout multiple locations (bed, living room, other rooms). Thus, in our level scale, we used as a criterion the location of bed bugs in the room, apartment, or building. Based on this scale of the level of infestation, most of the rooms inspected had an infestation level ranging from 0 to 2, followed by level 4 for the building. The originality of this scale, based on the distribution of the bed bugs in the room and in the dwelling as a whole, made it possible to map and visualise the location of these insects in the building and to understand how bed bugs move between rooms and floors in order to control this spread, adjust treatment according to the level, and monitor the infestation over time. During our inspection, bed bugs were collected in rooms that had been treated with insecticides, raising the suspicion of possible insecticide resistance in these samples. Indeed, insecticide resistance is a major problem in the fight against bed bugs^3^. In order to better control these pests, it would be interesting to investigate the mechanism of resistance, whether there has been genetic modification, and whether this mechanism is reversible. This is particularly interesting in light of one study which has been conducted describing pyrethroids, organophosphate, neonicotinoid, carbamate, chlorinated hydrocarbons, and phenyl pyrazole resistance^31^, which has been described in many countries and which has been identified as the primary catalyst for the transmission of bed bugs^32,33^. Most of the previous research involving homeless shelters has been performed in Canada and the USA^27,28^. Thus, our study represents the first documentation of abed bug infestation in a homeless shelter in France.

The difficulty of locating and identifying bed bugs has already been mentioned. Due to their small size and their dislike of light, they are occasionally mistaken for cockroaches and other arthropods. In order to identify a bed bug infestation, the area must be meticulously inspected, looking for obvious signs of current or past bed bug infestations, such as black spots (excreta of bed bugs) and bedbug exuviae^3^. In this study, we morphologically identified a single species, based on the adult criteria, the common bed bug (*C.lectularius*), known to be endemic in France^29^. It is important to indicate that species of the genus *Cimex* are difficult to distinguish with the naked eye. The task has become more difficult as specialists in the field become rarer. Molecular biology offers the possibility of perfect species identification, but it is time consuming, expensive and requires high-quality reference sequences^25^. In recent years, many research teams have recommended the use of MALDI TOF–MS to identify bacteria, parasites and arthropods, due to the accuracy of this tool^5,34^. It has proved useful in overcoming the limitations of the various methods of identifying different arthropods, and even different *Cimex* species, once a database has been established. To solve some of the problems in identifying the immature stages, extensive studies are currently underway to identify and differentiate between the different stages of laboratory-reared *Cimex* using mass spectrometry^21^.

During the blind test, we were able to correctly identify specimens down to the species level through straightforward dissection of the bed bugs’ heads and cephalothoraxes. Unexpectedly, 100% of the field-fresh bed bug MALDI TOF–MS analysis spectra were of high quality. The blind test results obtained from the adult head spectra showed a perfect match with the field-collected *C. lectularius* adults from Marseille, while the spectra obtained from immature cephalothoraxes matched perfectly with the laboratory-reared immature *C. lectularius* specimens.

When we compared our MALDI TOF–MS results with those in the Benkacimi study, which demonstrated that *C. lectularius* exhibits spectral variability with distinguishing peaks between males and females, we found that our results were inconsistent^5^. In our study, the PCA analyses were not able to distinguish between the MS spectra of male and female *C. lectularius* collected in the field. Given the environmental, geographic, and temporal factors, as well as arthropod-related microbiota since our samples are wild, fresh bed bugs, our results are comparable to those of Ndiaye, which presented no differences in bed bug spectra between male and female of the Senegalese species *C. hemipterus,* preserved in silica gel^22^.

Due to the sensitivity of MALDI TOF–MS, modifications in the protein profiles of objects may be brought about by sampling, preservation methods, dissection, and the selection of the part to be used, which varies depending on the arthropod and immature arthropod specimens^5,35^. Our sample consisted of two groups of immature specimens and adults, so we began with the head for adults and the cephalothorax for immature specimens, according to the Hamlili protocol^25^.

In our study, we detected a high carriage rate of *Wolbachia* endosymbionts, which are important in the reproduction or development of bed bugs. *Wolbachia* is a nutritional symbiont and is vertically transmitted by *C. lectularius,* partly because it is present in oocytes and is carried in a gonad-associated bacteriome^36^. Most hematophagous arthropods, including bed bugs, are unable to synthesise the eight essential B-vitamins that act as co-enzymes in metabolic processes to detoxify the heme and iron derived from the digestion of blood meals. A recent study underlined the nutritional importance of *Wolbachia* in bed bugs for vitamin B synthesis, heme detoxification, and bed bug development^37^. Our *Wolbachia* belong to two different clades: clade F, which has already been described in *C. lectularius*, and clade T, which, interestingly, has been recently described in* C. hemipterus*.

Although bed bugs are suspected to play a role in pathogen transmission, there have been no known cases of transmission anywhere in the world. Their ability to act as potential vectors has been demonstrated in experimental models^38,39^. In our study, the bed bug sample that tested positive for *C. burnetii* came from a room that had only a minor infestation and that had been treated with insecticide. This bacteria is responsible for Q fever, an infection with worldwide distribution, spread mainly by aerosol transmission^40^. In the 1960s, an experimental infection model reported that* C. burnetii* could be transmitted transstadially, regardless of the developmental stage of the experimentally infected bed bugs. *Coxiella burnetii* can multiply inside bed bugs, excrete itself in their faeces, and stay inside them for up to 250 days without losing its pathogenicity^4,41^. Our result is one more reason to raise awareness of the possible role of bed bugs, even anecdotal evidence, in the transmission of Q fever.

The metagenomic sequencing, has gained attention as effective tool to support One Health efforts. This approach helped in the detection of *rickettsial* sequence reads in a significant number of mosquitos pools^42^. This strategy is costly and also necessitates bioinformatics analysis^43^. It has been used on hematophagous arthropods such as mosquitoes, ticks, and lice^42,44^. Recently for the first time it has been employed in bed bugs in order to study bacterial communities^45^. As a result, while employing it, the pools must be prepared to enhance the concentration and load of microorganisms^46,47^. Pathogen identification in bed bugs has been studied using real-time PCR on a bacterial panel^22,25,21^. In our study, the presence of *Coxie*lla in a selectively positive sample prompted us to use a metagenomic method in the positive sample rather than relying on a pool.

Due to the emergence of insecticide resistance, bed bugs represent a contemporary challenge for infestation control and eradication. The priority is to employ mechanical controls, using vacuums, steam, diatomaceous earth, and tools that could be used by laypersons^3^. People working in the homeless shelter in our study have been trained in this method of control, and all beds have been treated with diatomaceous earth.

Homeless shelter infestations with bed bugs have been poorly investigated to date. Continuous monitoring and reporting of bed bug infestations is critical to fight bed bug infestations efficiently.

## Materials and methods

### Collection site and morphological identification of bed bugs

**Figure 5:**
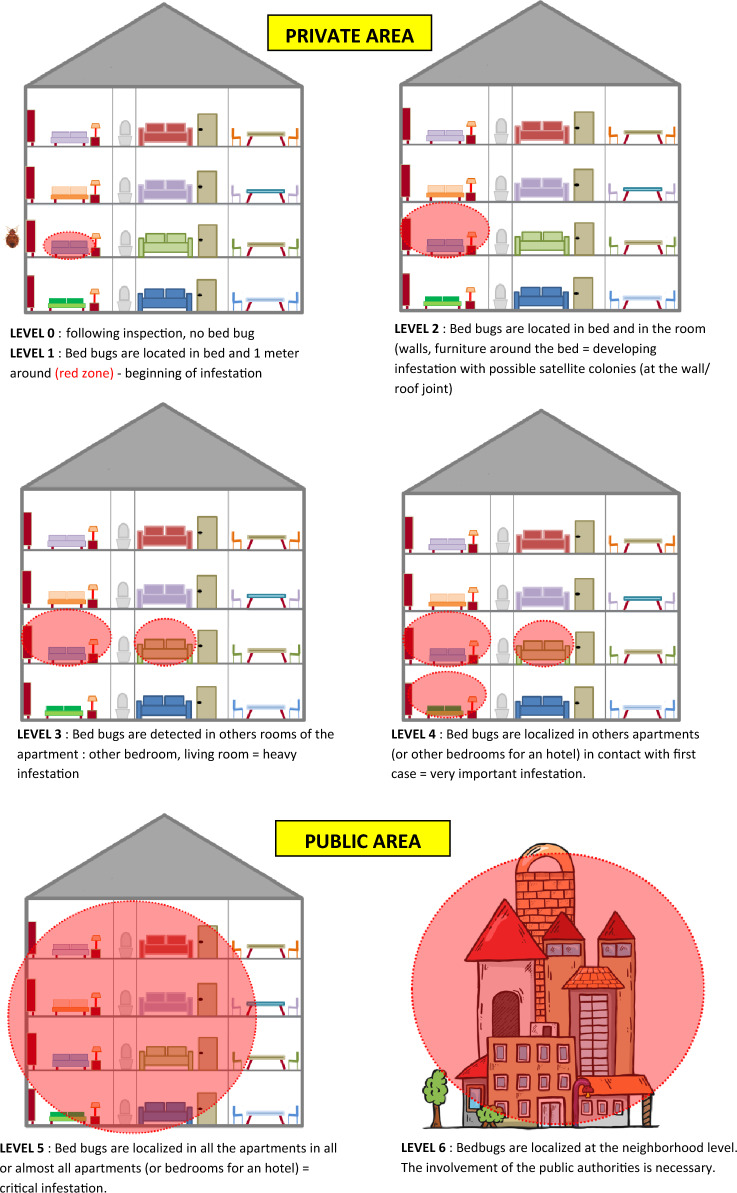
Assessment of seven bed bug infestation levels. Between levels 0 and 4, the infestations are in a “private area”. Levels 5 and 6 are in a “public area”.

Bed bugs were collected from a homeless shelter in Marseille in southern France in June 2022. The homeless shelter has a capacity to house 300 people. Only 68 rooms are permanently inhabited. After securing approval from the shelter manager and residents, each room was inspected for the presence of bed bugs, concentrating mainly on furniture, mattresses, bed frames, and wall nooks. The main warning signs of a bed bug infestation were dark faecal spots and the presence of exoskeletons on surfaces. To differentiate between extents of infestations, we developed seven bed bug infestation “levels” in order to create a common vocabulary between the professions and those confronted with the problem of bed bugs, and also to trigger a suitable control protocol according to the level of infestation. These seven levels (see Fig. 5) make it possible to immediately visualise the extent of the infestation. “Level 0” refers to an inspection that has confirmed there are no bed bugs present. “Level 1” means that bed bugs have been located in the bed and within one metre of it (red zone). This is the beginning of an infestation. The maximum level is “Level 6”, which means that bed bugs have been localised at the neighbourhood level, requiring the involvement of the public authorities. Between levels 0 and 4, the infestations are in a “private area”. Levels 5 and 6 are in “public areas” and represent a public health issue.

Bed bugs were collected in sterile containers, labelled according to the infested rooms. They were morphologically identified using a ZEISS Axio Zoom V16 based on taxonomic identification criteria previously published by Usinger and Walpole^48^. Due to a lack of an identification key for immature *Cimex*, in particular in order to differentiate between immature *C. lectularius* and *C. hemipterus* specimens, the identification of immature stages was based on significant physical traits relevant to adult specimens^21^. A few samples were then selected and photographed using a Canon MP-E65mm lens (France).

### Bed bug preparation for MALDI TOF–MS analysis

Each bed bug sample was rinsed in 70% ethanol, rinsed twice with distilled water, and dried with sterile filter paper^5^. Using a Leica ES2 stereomicroscope, the heads of adults and the cephalothoraxes (head and thorax) of immature samples were dissected using a new sterile blade for each sample. The cephalothoraxes from immature stages and the heads from adults were homogenised in 15 µl and in 40 µl of the extraction solution, respectively (70% formic acid and 50% acetonitrile) with glass beads (1.0 mm in diameter, BioSpec Products), using the TissueLyser device (Qiagen, Germany) at a frequency of 30 movements per second for three cycles of one minute^5^. One microlitre of the protein extract supernatant from each sample was spotted in quadruplicate on a MALDI TOF–MS steel target plate (Bruker Daltonics, Germany). The spots were left to dry at room temperature and then covered with one microlitre of matrix solution composed of saturated α-cyano-4-hydroxycinnamic acid (Sigma, Lyon. France), 50% acetonitrile, 2.5% trifluoroacetic acid and HPLC-grade water. The target plate was dried at room temperature before being inserted into the MALDITOF-MS instrument (Bruker Daltonics, Germany) for analysis. The remaining body parts were stored at 20 °C for molecular biology and further analysis^5,22,25^.

### MALDI TOF–MS parameters

The spectral profiles obtained from the bed bugs’ heads (adults) and cephalothoraxes (immature stage) were obtained using a Microflex LT MALDITOF mass spectrometer with FlexControl software (version 3.3, Bruker Daltonics). The spectra were acquired in a positive linear mode at a laser frequency of 50 Hz. The accelerating voltage was 20 kV, and the extraction delay time was 200 ns. Each spectrum corresponded to the ions obtained from the 240 laser shots performed in six regions at the same location and acquired automatically using the AutoXecute Flex Control software (version 2.4; Bruker Daltonics). The MS profiles were visualised using the FlexAnalysis v3.4 software, MALDI Biotyper Compass Explorer v4.1.70 (Bruker Daltonics), and ClinProTools v3.0 software (Bruker Daltonics) was used for data processing^49^.

### MALDI TOF–MS spectral analysis

All the spectral profiles obtained from the fresh bed bugs’ heads (adults) and cephalothoraxes (immature stage) were visualised using the FlexAnalysis v.3.3 software and ClinProTools 2.2 software (Bruker Daltonics) to evaluate spectral quality (smoothing, baseline subtraction, peak intensities). In order to determine the ability of the MALDITOF-MS tool to distinguish between male and female bed bugs, the MS spectra of 20 randomly selected specimens of both sexes were used for a principal component analysis (PCA) using ClinProTools 2.2^22,50^.

### Blind test for bed bug identification

Blind tests were performed by comparing the spectra of our bed bug specimens against our pre-existing database, which included spectra of *C. hirundinis*, *C. hemipterus*, and *C. lectularius* (adult and immature specimens) using MALDI-Biotyper software v3.0. (Bruker Daltonics)^5,22,25^. The LSVs obtained were used to assess the accuracy of species’ identification. For proper identification, the spectrum with the greatest log-score value [LSV] among the four spots was chosen^25^.

### DNA extraction and molecular identification of bed bugs

Bed bug DNA was extracted from the halves of each bed bug after overnight incubation at 56 °C in a 1.5 ml tube containing 180 µl of G2 lysis buffer and 20 µl of proteinase K (Qiagen, Hilden, Germany). The EZ1 DNA tissue kit (Qiagen) was used for DNA extraction in accordance with the instructions provided by the manufacturer. DNA from each sample was eluted with 100 µ of Tris–EDTA (TE) buffer (Qiagen), observed using Thermo Scientific NanoDrop 1000 (United States), and kept at − 20 °C until further analysis^25^.

### Bed bug bacterial detection

Quantitative real-time PCR (qPCR) was used to screen all bed bug DNA samples for the presence of *Borrelia* spp., *Coxiella burnetii*, *Rickettsia* spp., *Bartonella* spp., the bacteria of the Anaplasmataceae family, and *Wolbachia* spp. For real-time qPCR, the reaction mixture contained 5 µl of the DNA template and 15 µl of the reaction mix, as described previously^22^. Positive samples for *23S rRNA* Anaplasmataceae were subjected to a specific qPCR to screen for *Wolbachia,* targeting the *16S rRNAWolbachia* gene. The *IS1111* was used to identify *Coxiella burnetii*^51^, Positive samples were then confirmed by the second *IS30A* gene. Positive controls included the DNA of *Borrelia crocidurae*, *Anaplasma phagocytophilum*, *Coxiella burnetii*, *Rickettsia montanensis*, and *Bartonella elizabethae*. The PCR mix alone was employed as a negative control^52^. When the qPCR assays allowed amplification of the two separate *C. burnetii* specific genes, the sample was considered positive^53^. Only specimens with a cycle threshold (Ct) lower than 35 were considered positive^22^. In order to identify the most genetically similar *Wolbachia* genotypes using the 16 s RNA gene, more than 10% of the qPCR-positive samples for *Wolbachia* spp. were randomly chosen to be sequenced, assembled, and compared to GenBank by BLAST searches. Identification of positive samples by standard PCR was based on amplification and sequencing, as previously described^5,54^. Amplification was confirmed by electrophoresis on a 1.5% agarose gel and PCR products were purified using NucleoFast 96 PCR plates (Macherey–Nagel, Hoerdt, France) according to the manufacturer’s instructions. Amplicons were sequenced using the Big Dye Terminator Cycle Sequencing Kit (Perkin Elmer Applied Biosystems, Foster City, CA) and the resulting sequences were assembled using ChromasPro software (ChromasPro 1.7, Technelysium Pty Ltd., Tewantin, Australia).

The assembled and trimmed sequences were submitted for comparison to the GenBank database by NCBI BLAST search (http://blast.Ncbi.nlm.nih.gov/Blast.cgi). We used MEGA software version 7.0.21 to perform our sequence alignments and construct phylogenetic trees with 1000 bootstrap replications for the *16S rRNA* gene. The qPCR and standard PCR tests were performed using primers and probes listed in Supplementary data 1^22^.

### Metagenomic (Illumina Miseq) analysis of bed bugs positive for *Coxiella burnetii*

Positive DNA from *C. burnetii* were quantified with a Qubit assay using the high sensitivity kit from Life Technologies, Carlsbad, CA, USA, at a concentration of 0.2 ng/l, before being subjected to whole genome sequencing. The next step was barcoding and pairwise sequencing of the genomic DNA using MiSeq technology (Illumina Inc., San Diego, CA, USA). Dilution was performed to prepare the paired-end library, which required 1 ng of each genome as input. The “tagging” step fragmented and tagged the DNA. Cycle-limited PCR amplification (12 cycles) then completed the tagging adapters and introduced the dual-index barcodes. After being purified on AMPure XP beads (Beckman Coulter Inc., Fullerton, CA, USA), the libraries were normalised on beads in accordance with the Nextera XT protocol (Illumina). Normalised libraries were pooled into a single library for sequencing on the MiSeq. The flow cell and the pooled single-strand library were then loaded onto the instrument and the reagent cartridge. In a single 39-h run, 2 × 250 bp automated cluster generation and paired-end sequencing with dual index reads were completed^55,56^.

## Supplementary Information

Supplementary Information 1.

Supplementary Information 2.

Supplementary Information 3.

## Data Availability

The data presented in this study (sequences) are openly available in GenBank (National Centre for Biotechnology Information, NCBI) under the following accession numbers: OP740254 to OP740259.
