# Supplementary material for: Survey of bed bug infestations in homeless shelters in southern France
Source: Sci Rep. 2023 Aug 2;13:12557. doi: 10.1038/s41598-023-38458-2 (PMC10397270; doi:10.1038/s41598-023-38458-2)
Supplement: Supplementary file 1 — Supplementary Information 1. [file 41598_2023_38458_MOESM1_ESM.docx]

**Table 1:** Oligonucleotide sequences of primers and probe used in the study for qPCRs and conventional PCRs

| System name | Assay specificity | Targeted gene | | Primer sequences | | Reference | | | |  |
| --- | --- | --- | --- | --- | --- | --- | --- | --- | --- | --- |
| Real Time  Quantitative PCR | *Borrelia* sp. | *ITS4* | Bor_ITS4_ F (5’-GGCTTCGGGTCTACCACATCTA-3’)  Bor_ITS4_ R (5’-CCGGGAGGGGAGTGAAATAG-3’)  Bor_ITS4_ P (6FAM-TGCAAAAGGCACGCCATCACC) | | | | | (Diarra et al. 2020) | | |
|  | *Bartonella* sp. | *ITS2* | Barto_ITS2_F (5’- GGGGCCGTAGCTCAGCTG-3’)  Barto_ITS2_R (5’- TGAATATATCTTCTCTTCACAATTTC-3’)  Barto_ITS2_P (6FAM- CGATCCCGTCCGGCTCCACCA) | | | | | (Diarra et al. 2020) | | |
|  | Anaplasmatacae | *23S rRNA* | TtAna_F (5’-TGACAGCGTACCTTTTGCAT-3’)  TtAna_R (5’- GTAACAGGTTCGGTCCTCCA-3’)  TtAna_P (6FAM- GGATTAGACCCGAAACCAAG) | | | | (Dahmani et al. 2015) | | | |
|  | *Coxiella burnetti* | *ISS11* | CB_IS1111_0706F (5’-CAAGAAACGTATCGCTGTGGC-3’)  CB_IS1111_0706R (5’-CACAGAGCCACCGTATGAATC-3’)  CB_IS1111_0706P (6FAM-CCGAGTTCGAAACAATGAGGGCTG) | | | | | | (Diarra et al. 2020) | |
|  |  | *IS30A* | CB_IS30A F (5’-CGCTGACCTACAGAAATATGTCC-3’)  CB_IS30A R (5’-GGGGTAAGTAAATAATACCTTCTGG-3’)  CB_IS30A P (5’-CATGAAGCGATTTATCAATACGTGTATGC-3’) | | | | | | Mediannikov et al.2010 | |
|  | *Rickettsia* | *RKND* | RKND03_F (5’-GTGAATGAAAGATTACACTATTTAT-3’)  RKND03_R (5’-GTATCTTAGCAATCATTCTAATAGC-3’)  RKND03_R (6FAM- CTATTATGCTTGCGGCTGTCGGTTC) | | | | | (Diarra et al. 2020) | | |
|  | *Wolbachia* | *16S rRNA* | Wol-301-F (5’-TGGAACTGAGATACGGTCCAG-3’)  Wol-478-R (5’-GCACGGAGTTAGCCAGGACT-3’)  Wol-347-P (6FAM-AATATTGGACAATGGGCGAA) | | | | | (Laidoudi, et al. 2020) | | |
|  | *Wolbachia* | *16S rRNA* (rrs) | W-Spec_F (5’-CATACCTATTCGAAGGGATAG-3’)  W-Spec_R (5’-AGCTTCGAGTGAAACCAATTC-3’) | | (Djiba et al. 2013) | | | | | |
