# Supplementary figures and images for: Survey of bed bug infestations in homeless shelters in southern France
Source: Sci Rep. 2023 Aug 2;13:12557. doi: 10.1038/s41598-023-38458-2 (PMC10397270; doi:10.1038/s41598-023-38458-2)

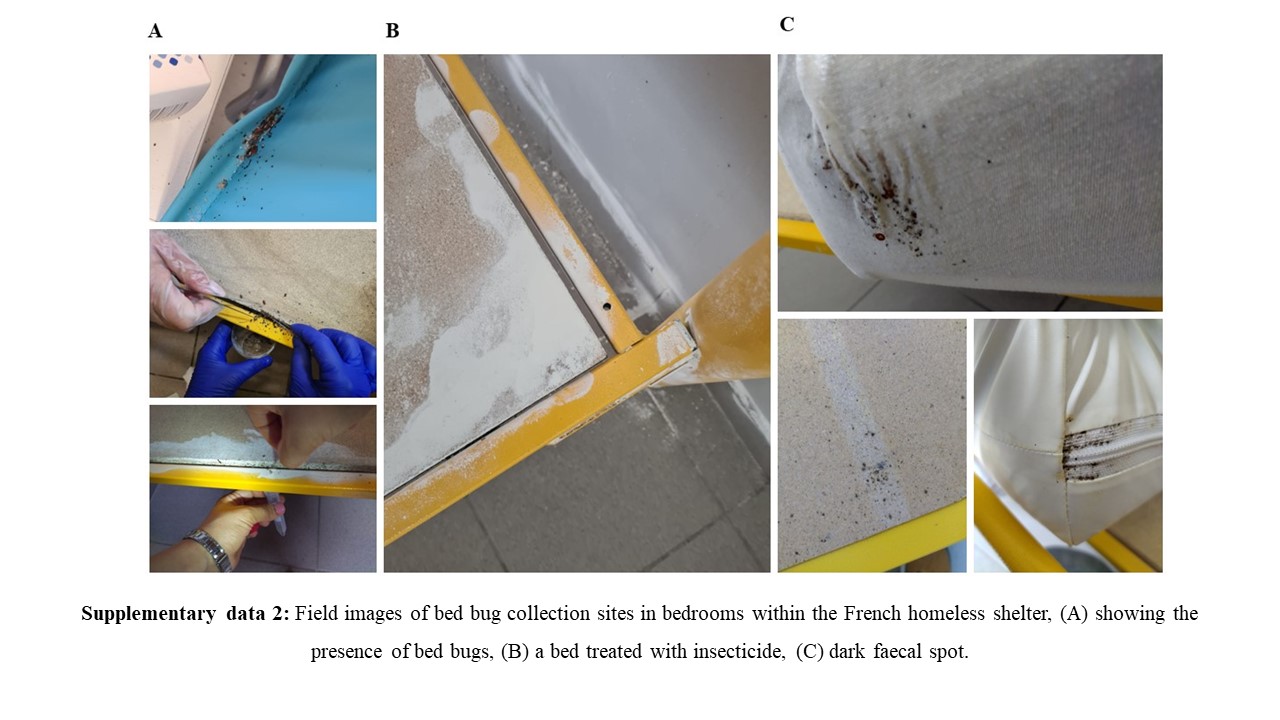

Supplement: Supplementary file 2 — Supplementary Information 2. [file 41598_2023_38458_MOESM2_ESM.jpg]

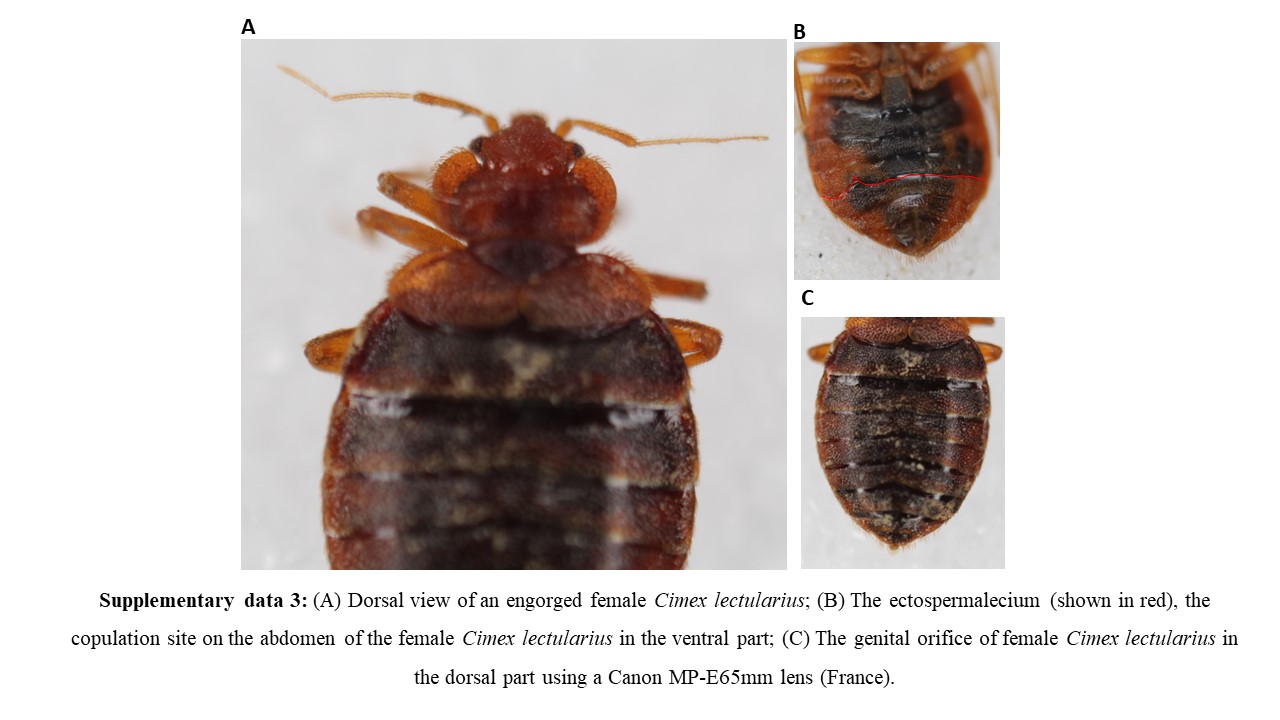

Supplement: Supplementary file 3 — Supplementary Information 3. [file 41598_2023_38458_MOESM3_ESM.jpg]
